# Clinical characteristics, treatment regimen and duration of hospitalization among COVID-19 patients in Ghana: a retrospective cohort study

**DOI:** 10.11604/pamj.supp.2020.37.1.25718

**Published:** 2020-09-15

**Authors:** Mary Eyram Ashinyo, Vida Duti, Stephen Dajaan Dubik, Kingsley Ebenezer Amegah, Selorm Kutsoati, Ebenezer Oduro-Mensah, Peter Puplampu, Martha Gyansa-Lutterodt, Delese Mimi Darko, Kwame Ohene Buabeng, Anthony Ashinyo, Anthony Adofo Ofosu, Nyonuku Akosua Baddoo, Samuel Kaba Akoriyea, Francis Ofei, Patrick Kuma-Aboagye

**Affiliations:** 1 Institutional Care Division, Ghana Health Service Headquarters, Private Mail Bag, Accra, Ghana,; 2 IRC-Ghana, Cantoments-Accra, Ghana,; 3 School of Allied Health Sciences, University for Development Studies, Tamale, Ghana,; 4 Department of Health Information, Hohoe Municipal Hospital, Hohoe, Ghana,; 5 Ga East Municipal Health Directorate, Accra, Ghana,; 6 Ga East Municipal Hospital, Accra, Ghana,; 7 Department of Medicine, Korle Bu Teaching Hospital, Accra, Ghana,; 8 Technical Coordination Directorate, Ministry of Health, Accra, Ghana,; 9 Food and Drug Authority, Accra, Ghana,; 10 Department of Pharmacy Practice, Kwame Nkrumah University of Science and Technology, Ghana,; 11 National AIDS/STI Control Programme, Accra, Ghana,; 12 Ghana Health Service, Accra, Ghana,; 13 National AIDS/STI Control Programme, Ghana Health Service, Accra, Ghana,; 14 Department of Medicines, School of Medicine and Dentistry, University of Cape Coast,; 15 Office of the Director General, Ghana Health Service Headquarters, Accra, Ghana

**Keywords:** COVID-19, clinical characteristics, azithromycin, chloroquine, hydroxychloroquine, supportive treatment, duration of hospitalization, infectious diseases, Ghana

## Abstract

**Introduction:**

COVID-19 is a global pandemic seen in modern times. The clinical characteristics, treatment regimen and duration of hospitalization of COVID-19 patients remain unclear in Ghana.

**Methods:**

we retrospectively reviewed the secondary data of 307 discharged COVID-19 patients to characterize their demographics, clinical symptoms, treatment regimen given and duration of hospitalization.

**Results:**

the mean age and temperature of the patients were 37.9 years and 36.3°C, respectively. The majority (85.7%) of the cases reviewed were asymptomatic; for those presenting with symptoms, the main ones were cough (50%), fever (29.6%), headache (27.3%), and sore throat (22.7%). Comorbidities were present in 25.1% of the patients; the popularly reported comorbidities were hypertension (71.4%), asthma (7.8%) and diabetes (6.5%). The average duration of hospitalization was 13.8 days, and the duration of hospitalization for patients managed with azithromycin + chloroquine (AZ+CQ) was 10.4 days, followed closely by those managed with hydroxychloroquine (HCQ) only, 11.0 days. There was longer duration of hospitalization among patients who received AZ only compared to patients receiving AZ + CQ (3.24 ± 1.10 days, p=0.037; 95% CI 0.11, 6.37). Linear regression analysis showed that the duration of hospitalization for patients who received AZ only was 2.7 days, which was higher than that of patients who received AZ+CQ and HCQ only (95% CI 0.44, 4.93; p=0.019).

**Conclusion:**

in this cohort of COVID-19 patients, the common symptoms were cough, fever, headache, and sore throat. The use of AZ+CQ or HCQ only as a therapy for managing COVID-19 patients shortened the duration of hospitalization.

## Introduction

The novel coronavirus disease 2019 (COVID-19), which originated in Wuhan, China in December 2019, has spread to almost every country across the globe (WHO, 2019) [1]. Since the first report of COVID-19 infection in China, the WHO (10: 00 CEST, 28 July 2020) has notified 16,341920 laboratory-confirmed cases of COVID-19 from more 200 countries with 650,805 deaths (case fatality rate of 4%) [2]. As of 28 July 2020, there are 33,624 laboratory-confirmed COVID-19 virus infections in Ghana and 168 COVID-19-related deaths (approximate case fatality rate of 0.5%), which is far lower than the global case fatality rate [3]. The COVID-19 pandemic has forced researchers across the globe to scramble for possible strategies to interrupt its transmission and for effective management of the disease. Consequently, several clinical trials have been initiated in an attempt to find effective therapeutic treatment and prevention for COVID-19 [4]. While several clinical trials are ongoing, HCQ, CQ and AZ have gained more attention than any other medication in managing COVID-19 cases across the globe [4-8]. Evidence from studies suggests the ability of HCQ and CQ with the possible addition of AZ to suppress the activity of severe acute respiratory syndrome coronavirus 2 (SARS-COV-2) [9,10]. Indeed, the combination of HCQ and AZ in an open-label non-randomized clinical trial is associated with viral clearance in COVID-19 patients [10]. In Ghana, HCQ or CQ, with a possible combination of CQ+AZ or HCQ+AZ, is used as the first-line treatment in managing asymptomatic and mild/moderate symptomatic COVID-19 cases [11]. Prior to the use of HCQ or CQ as first-line treatment, the Food and Drug Authority (FDA) of Ghana gave an emergency authorization based on the guidelines for emergency use of medicinal products [12]. Previous studies have examined the effect of HCQ and AZ usage on mortality reduction in hospitalized COVID-19 patients and described the clinical features of COVID-19 patients in specific countries [5,7,8], but there are still gaps in knowledge and the understanding of COVID-19 regarding the patient´s clinical characteristics, treatment regimen and duration of hospitalization in Ghana. This retrospective study was conducted to describe the presenting clinical features of COVID-19 patients, treatment regimen and duration of hospitalization in two treatment centres in Ghana.

## Methods

We conducted a retrospective study in two designated COVID-19 treatment centres in the Greater Accra region of Ghana, which is regarded as the country´s epicentre, namely, Ga East Municipal Hospital and Pentecost Convention Centre. All patients admitted to the treatment centres were laboratory-confirmed cases of COVID-19. Included in our review were the records of all available COVID-19 patients who were discharged between 23 March to 29 June 2020. During this period, all confirmed cases of COVID-19 were discharged based on full recovery. A team of public health officers, under the supervision of the principal investigator, extracted the data from patients´ records after receiving appropriate training. Folders with incomplete records were excluded from the review. In all, 307 discharged COVID-19 patient records were reviewed at the two treatment centres. Data were obtained on the following from the discharged COVID-19 cases: age, sex, date of admission, date of discharge, temperature on admission; temperature greater than 37.2 was defined as fever, syndromic categorization (symptomatic or asymptomatic), severity of COVID-19 infection, clinical features, comorbidities, outcome of admission post therapy (death/discharge), type of discharge (full recovery/home treatment plan), treatment given during hospitalization (HCQ, CQ, AZ, vitamin C, etc. and other medications for comorbidities the patients had).

**Exposures:** based on the available data, the patients were categorized into five groups based on the type of treatment they received during hospitalization as follows: (1) CQ+AZ, (2) HCQ+AZ, (3) AZ only, (4) HCQ only and (5) Supportive treatment only; the latter refers to patients who received vitamin C and analgesics (paracetamol or ibuprofen). All the treatments were in line with Ghana´s Provisional COVID-19 Standard Treatment Guidelines (STG).

**Outcome measures:** the outcome variable for the study was duration of hospitalization. A patient was considered fully recovered if the patient test negative to the COVID-19 virus on two consecutive PCR assay results with samples taken at least twenty-four hours apart. The duration of hospitalization was calculated by subtracting the date of admission from the date of discharge, while the average duration of hospitalization for the study participants was calculated by dividing the total length of stay by the total number of discharges for the same period. Patients discharged based on a home treatment plan were excluded from the final analysis. Home treatment plans refer to patients who were discharged to be managed at home, i.e., those who were discharged based on patient request and if the patient home is assessed to be safe.

**Statistical analysis:** data were analysed using STATA 14.2. Descriptive statistics were presented as mean and standard deviation for continuous variables and frequencies (percentages) for categorical variables. One-way analysis of variance (ANOVA) was conducted to determine if there was any significant differences in the duration of hospitalization across the five different treatment options. Finally, a linear regression was conducted to ascertain the relationship between the treatment regimen and duration of hospitalization while controlling for patient gender, age and comorbidity. All analyses were considered significant at a p-value less than 0.05 at a confidence interval of 95%.

**Ethical consideration:** the study was approved by the Ghana Health Service Ethics Review Committee. Permission for the use of the data in this study was granted by Ghana Health Service.

## Results

**Patient demographic and presenting clinical characteristics:** patient characteristics, syndromic categorization, comorbidities and outcomes of discharge are presented in Table 1. The mean age of the COVID-19 patients was 37.9 years (standard deviation = 16.3) with most (29.6%) of them within the age group 31-44. Most (56.7%) of the cases were males. The mean presenting temperature on admission was 36.3°C, ranging from 33.4 - 40.8°C. On average, the duration of hospitalization was 13.8 days and the majority (85.7%) of the cases reviewed were asymptomatic COVID-19 infections. The prevalence of comorbidities was 77 (25.1%). The common comorbidities were hypertension (55, 71.4%), followed by asthma (6, 7.8%) and diabetes mellitus (5, 6.5%). Almost 80% of the cases were discharged based on full recovery, while all of the patients were discharged alive. The presenting clinical symptoms of the COVID-19 cases are presented in Table 1. The most common clinical symptoms were cough (22, 50%), fever (13, 29.6%), headache (13, 27.3%), sore throat (10, 22.7%) and nausea (5, 11.4%). The least common symptoms among the cases reviewed were chest pain, chills, fatigue, shortness of breath, vomiting, skin rash, joint ache and loss of appetite.

**Table 1:** patient characteristics, syndromic categorization, comorbidities and outcomes of discharge

Independent Variables	Mean	SD	Min	Max
Age	37.86	16.31	1	83
Temperature on admission	36.27	0.72	33.4	40.8
Duration of hospitalization	13.82	3.47	5	22
	**Frequency, N = 307**		**Per cent (%)**	
**Age Group (In years)**				
< 18	26		8.5	
18-30	89		29.0	
31-44	91		29.6	
45-64	82		26.7	
≥ 65	19		6.2	
**Gender**				
Female	133		43.3	
Male	174		56.7	
**Syndromic**				
Symptomatic	44		14.3	
Asymptomatic	263		85.7	
**Any Comorbidity**				
No	230		74.9	
Yes	77		25.1	
**Comorbidities**				
Asthma	6		7.8	
Diabetes Mellitus	5		6.5	
Hypertension	55		71.4	
Hypertension & Diabetes Mellitus	11		14.3	
**Type of Discharge**				
Full Recovery	244		79.5	
Home treatment plan	63		20.5	
**High temperature (⁰C)**				
37.3-38.0	9		69.2	
38.1-39.0	2		15.4	
> 39.0	2		15.4	
Baseline Clinical Characteristics*				
Fever	13		29.6	
Cough	22		50.0	
Headache	12		27.3	
Sore throat	10		22.7	
Nausea	5		11.4	
Chest pain	3		6.8	
Chills	3		6.8	
Fatigue	3		6.8	
Diarrhoea	2		4.6	
Loss of appetite	2		4.6	
Muscle ache	2		4.6	
Skin rash	1		2.3	
Joint ache	1		2.3	

* Multiple response variables

**Figure 1:**
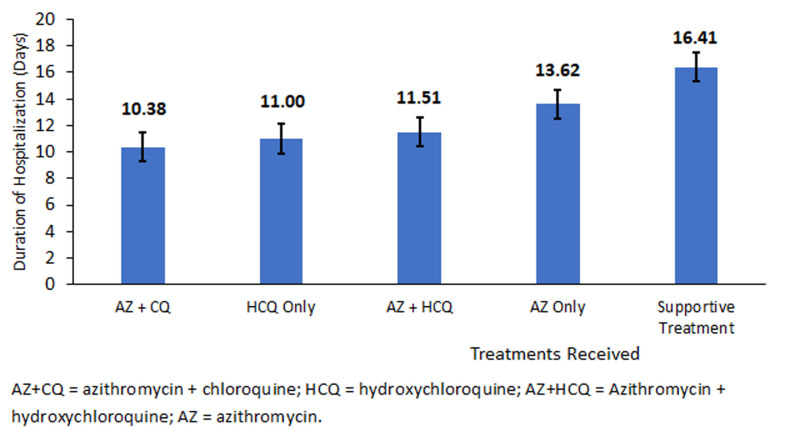
duration of hospitalization by treatment received

**Duration of hospitalization by treatment option:** females (14.2 days) had a long duration of hospitalization compared to males (13.6 days). As seen in figure 1, the duration of hospitalization for COVID-19 patients who received AZ+CQ was 10.4 days, followed by those who received HCQ (11.0 days). Patients on supportive treatment had a longer length of hospitalization of 16.4 days. As seen in Table 2, analysis of variance (ANOVA) showed that there was a significant difference between treatment options as determined by one-way ANOVA (F (4, 239) = 19.74, p<0.001). A Bonferroni post hoc test revealed that patients receiving AZ only had a longer length of stay in the hospital compared to patients receiving AZ + CQ (3.24 ± 1.10 days, p=0.037) and a longer length of stay in the hospital among patients who received AZ only compared to patients who received AZ + HCQ (2.10 ± 0.55 days, p<0.002). COVID-19 patients who received supportive treatment spent 6.03 more extra days in the hospital than patients who received AZ+CQ (6.03 ± 1.14 days, p<0.001). There were no significant differences between the duration of hospitalization of patients who received AZ+HCQ compared to AZC+CQ (1.14 ± 1.18 days, p=1.000) or HCQ only compared to AZ+CQ (0.63 ± 2.05 days, p=1.000).

**Table 2:** pairwise comparison of means differences between duration of hospitalization and treatment options

Treatment option	Difference of Mean	SE of Difference	T Value	95% CI	Adjusted p-value
AZ+HCQ vs. AZ+CQ	1.14	1.18	0.97	-2.20-4.48	1.000
AZ Only vs. AZ+CQ	3.24	1.10	2.93	0.11-6.37	0.037
HCQ Only vs. AZ+CQ	0.63	2.05	0.30	-5.19-6.44	1.000
Supportive Rx vs. AZ+CQ	6.03	1.14	5.28	2.79-9.27	< 0.001
AZ Only vs. AZ+HCQ	2.10	0.55	3.81	0.54-3.66	0.002
HCQ Only vs. AZ+HCQ	-0.51	1.82	-0.28	-5.66-4.64	1.000
Supportive Rx vs. AZ+HCQ	4.89	0.63	7.82	3.12-6.67	< 0.001
HCQ Only vs. AZ Only	-2.61	1.77	-1.48	-7.63-2.40	1.000
Supportive Rx vs. AZ Only	2.79	0.47	5.90	1.45-4.13	< 0.001
Supportive Rx vs. HCQ Only	5.41	1.80	3.01	0.32-10.49	0.029

AZ+HCQ = azithromycin + hydroxychloroquine; AZ+CQ = azithromycin + chloroquine; AZ = azithromycin; HCQ = hydroxychloroquine; AZ+HCQ = azithromycin + hydroxychloroquine; RX = treatment

**Relationship between duration of hospitalization and treatment option received:** a linear regression analysis while controlling for age, gender, and comorbidities is presented in Table 3. The duration of hospitalization on AZ only was 2.68 days longer than that of patients who received AZ+CQ as well as those who received HCQ only. For patients who were treated on supportive treatment, the predicted duration of hospitalization from COVID-19 was 5.59 days higher than patients who were treated with AZ+CQ or HCQ only.

**Table 3:** relationship between duration of hospitalization and treatment received

Duration of hospitalization	Estimate	SE	t	p-value	95% CI
**Treatment Option**					
AZ+HCQ	0.69	1.21	0.57	0.570	-1.70 - 3.09
AZ Only	2.68	1.14	2.36	0.019	0.44 - 4.93
HCQ Only	0.24	2.07	0.12	0.906	-3.83 - 4.32
Supportive Rx	5.59	1.18	4.74	< 0.001	3.27 - 7.91

AZ+HCQ = Azithromycin + hydroxychloroquine; AZ = azithromycin; HCQ = hydroxychloroquine; RX = treatment. Number of observations = 244; F (10, 233) = 8.85; Prob > chi2 = 0.0000; R-squared = 0.2691

## Discussion

In this study, we aimed to describe the characteristics of patients with COVID-19 and determine the association between treatment with hydroxychloroquine or azithromycin and the duration of hospitalization in two COVID-19 designated treatment centres in Ghana. The mean age of the patients with COVID-19 in the two treatment centres was 37.9 years with minimum and maximum ages of 1 and 83, respectively. These findings showed that COVID-19 disproportionately infects the younger and economically viable population in Ghana. Our outcome is similar to a previous study conducted in Saudi Arabia [13] but varies from studies conducted in Beijing; mean age of 58 [14], Wuhan China; mean age of 63 [15], mean age of 56 [16], and China; mean age of 47 [17]. Males (56.7%) were more affected than females; this is comparable to other studies across the globe [14,18-21]. Gender is a risk factor for COVID-19 severity, with more males being infected with the global pandemic [21]. The vast majority of the COVID-19 patients we assessed were asymptomatic. This varies from previous studies that reported most symptomatic cases [19,22]. This has implications for the ability of public health authorities to detect and contain the virus early in Ghana. Asymptomatic cases have the potential to spread the COVID-19 virus widely and silently through human populations [23]. Perhaps mass testing could identify most of the asymptomatic cases, thereby helping in the interruption of transmission of the COVID-19 virus in Ghana. The mean presenting temperature on admission was 36.3°C, ranging from 33.4 - 40.8°C, disagreeing with studies from the literature [24,25]. Perhaps temperature screening in most Ghanaian institutions as a measure to identify suspected COVID-19 cases may be missing a more significant number of suspected cases. Higher body temperature among COVID-19 patients impacts mortality [24]. Interestingly, COVID-19 patients presenting with lower body temperature have an increased risk of mortality [24].

The most common clinical symptoms were cough, fever, headache, and sore throat. In other jurisdictions, commonly reported clinical symptoms among COVID-19 patients are cough, fever, fatigue, and headache [14,19,26-28]. The prevalence of comorbidities among COVID-19 patients was 25.1%. Common comorbidities were hypertension, asthma and diabetes mellitus. This matches the prevalence of comorbidities reported in previous studies [14,21]. Hypertension and diabetes mellitus are commonly reported comorbidities among COVID-19 patients [7,21,22,29,30]. The COVID-19 virus is severe among people with underlying medical conditions and is associated with poor clinical outcomes [30,31]. In this study, we report an average duration of hospitalization of 13.8 days, which is comparable to what has been published by Zhao *et al*. in Beijing [32] and a systematic review by Rees *et al*. [33]. Wang and colleagues reported shorter hospitalization times (10 days) for COVID-19 patients in Wuhan, China [34]. Our finding is shorter than what has been reported in a group of COVID-19 patients in Vietnam by 2.2 days [35] and in SARS patients by 3.6 days [36]. A longer duration of hospitalization can potentially overwhelm the health systems, including the healthcare workforce. In the case of infectious diseases such as COVID-19, longer hospitalization can promote transmission to healthcare workers and to the general population.

Our analysis showed that the average duration of hospitalization was shorter in patients who received AZ+CQ (10.38 days) and HCQ only (11.0 days). Comparing mean differences by treatment options revealed that patients who received AZ only were more likely to spend 3.24 and 2.10 extra days in the hospital compared to patients who received AZ +CQ and AZ+HCQ, respectively. While controlling for age, gender and comorbidities, the predicted duration of hospitalization among patients who received AZ only was 2.68 days, higher than patients who received AZ+CQ. These findings imply that COVID-19 patients managed with AZ+CQ tend to spend fewer hospital stays. However, there were no significant differences between the duration of hospitalization of patients managed with AZ+CQ and those managed with HCQ only. The activity of CQ and HCQ is the same since their mechanism of action is similar [9]. Findings from Gautret and colleagues suggest that HCQ treatment is associated with viral clearance in COVID-19 patients, which is reinforced by AZ [10]. A retrospective analysis in Marseille, France showed that patients treated with HCQ+AZ have better clinical outcomes than other treatment options [37]. In New York State, treatment with HCQ, AZ or both compared with neither therapy did not influence in-hospital mortality [6]. This study is a descriptive study. Randomized clinical control trials are recommended to validate the findings in this study.

## Conclusion

In this cohort of COVID-19 patients assessed, the majority were asymptomatic. The few with symptoms that were commonly reported include cough, fever, headache and sore throat. Based on the data of 244 fully recovered patients, the use of the AZ+CQ combination or HCQ alone shortened the duration of hospitalization compared to the use of AZ only or supportive treatment. This supports the recommendation in the Ghana standard treatment guidelines that if not contraindicated, HCQ or CQ should be used efficiently alone or in combination with AZ to optimize the management of asymptomatic and mild cases of COVID-19.

### What is known about this topic

- COVID-19 has been described as a global public health emergency seen in modern times;
- As of 28 July 2020, there were 33,624 laboratory-confirmed COVID-19 virus infections and 168 COVID-19-related deaths in Ghana, an approximate case fatality rate of 0.5%;
- Currently, the clinical characteristics, treatment regimen and duration of hospitalization of COVID-19 patients remain unknown in Ghana.

### What this study adds

- A vast majority of the COVID-19 patients were asymptomatic, with few presenting with symptoms of cough, fever, headache, sore throat, nausea and chest pain;
- Comorbidities were present in 25.1% of the COVID-19 patients, with common comorbidities being hypertension, asthma and diabetes;
- The use of AZ+CQ or HCQ alone as a therapy for managing COVID-19 patients shortened the duration of hospitalization when compared to AZ alone or supportive treatment.
